# Wide-Temperature-Range Tachometer Based on a Magnetoelectric Composite

**DOI:** 10.3390/s25030829

**Published:** 2025-01-30

**Authors:** Boyu Xin, Qianshi Zhang, Lizhi Hu, Anran Gao, Chungang Duan, Zhanjiang Gong, Erdong Song, Likai Sun, Jie Jiao

**Affiliations:** 1 Key Laboratory of Polar Materials and Devices (MOE), Shanghai Center of Brain-Inspired Intelligent Materials and Devices, Department of Electronics, East China Normal University, Shanghai 200241, China; 2 School of Materials Science and Engineering, Nanjing University of Science and Technology, Nanjing 210094, China; 3 The 49th Research Institute of China Electronics Technology Group Corporation, Harbin 150001, China; 4 Shanghai Institute of Ceramics, Chinese Academy of Sciences, Shanghai 201800, China

**Keywords:** magnetoelectric composite, tachometer, rotational speed measuring, extreme temperature sensing

## Abstract

In this work, a tachometer based on a Metglas/PZT/Metglas magnetoelectric (ME) composite was developed to achieve high-precision rotational speed measurement over a wide temperature range (−70 °C to 160 °C). The tachometer converts external magnetic signals into electrical signals through the ME effect and operates stably in extreme temperature environments. COMSOL Multiphysics software was used for simulation analysis to investigate the ME response characteristics of the composite in such environments. To evaluate the properties of the ME composite under different conditions, its response characteristics at various frequencies, DC bias, and temperatures were systematically investigated. A permanent magnet and a DC motor were used to simulate gear rotation, and the voltage output was analyzed by adjusting the position between the sensor and the DC motor. The results show that the measured values of the ME tachometer closely match the set values, and the tachometer demonstrates high measurement accuracy within the range of 480 to 1260 revolutions per minute (rpm). Additionally, the properties of the ME composite at different temperatures were examined. In the temperature range from −70 °C to 160 °C, the ME coefficients exhibit good regularity and stability, with the measured trend closely matching the simulation results, ensuring the reliability and accuracy of the ME tachometer. To verify its practicality, the measurement capability of the ME tachometer was comprehensively tested under extreme temperature conditions. The results show that in high-temperature environments, the tachometer can accurately measure speed while maintaining a high signal-to-noise ratio (SNR), demonstrating excellent anti-interference ability. The proposed ME tachometer shows promising application potential in extreme temperature conditions, particularly in complex industrial environments that require high reliability and precision.

## 1. Introduction

With the continuous advancement of modern industrial technology, the demand for high-precision and highly reliable sensors has been growing steadily. Tachometers, as core components in mechanical systems, play an indispensable role in industrial automation, automotive manufacturing, and aerospace applications [1,2,3,4]. Traditional tachometers commonly utilize principles such as optics, the Hall effect, or the magnetoresistance effect [5,6,7,8]. These technologies meet the requirements for conventional applications but still face numerous challenges in complex working environments and extreme temperature conditions, such as performance instability, poor durability, and high maintenance costs. Therefore, the development of novel, high-performance, and low-cost tachometers for extreme temperature environments has become of paramount importance. In recent years, magnetoelectric (ME) composites have demonstrated great potential in the field of sensors due to their unique ME effect and excellent physical properties [9,10,11,12,13].

ME composites can convert external magnetic signals into electrical signals [14], offering advantages such as high sensitivity, fast response, and good stability. When applied to tachometers for non-contact measurement, these composites can significantly improve durability and reliability. In contrast, traditional mechanical tachometers rely on components such as gears and bearings, which wear out over time. In extreme temperatures and high-vibration environments, this wear becomes even more severe, leading to measurement errors, reduced sensitivity, and a shortened lifespan. Due to the unique non-contact working principle, which relies on the magnetostrictive and piezoelectric effects, ME composites can maintain stable operation across a much wider temperature range compared to traditional tachometers. Non-contact measurement improves performance stability, especially in complex environments involving extreme temperatures, high vibrations, and harsh industrial conditions. Furthermore, the main components of the ME tachometer are PZT ceramics and Metglas alloys, both of which are cost-effective. The simple composition and structure are advantageous for large-scale manufacturing and widespread applications.

These characteristics make ME composites an ideal choice for the development of tachometers based on novel principles. In 2007, Myers et al. reported a tachometer based on an ME composite [15], and confirmed the feasibility of using ME composites for speed measurement in high-temperature conditions. They selected PZT [Pb(Zr_x_Ti_1-x_)O_3_] with high Curie temperatures (325–340 °C) and Galfenol materials (Curie temperature over 500 °C) to ensure stability under high-temperature and high-pressure conditions. Although this combination expanded the working temperature range of the tachometer, the magnetostrictive behavior of Galfenol is highly anisotropic, requiring single-crystal or textured materials for optimal performance, which increases the cost and complexity of fabricating the ME composite. In 2016, Wu et al. proposed an ME angle sensor for rotational parameter detection [16], which consisted of an ME composite and a multi-pole magnetic ring. This research demonstrated excellent performance in dynamic testing environments and was capable of determining the position through phase shifts and sensing the eccentricity of rotating part. In 2018, Shi et al. reported a tachometer based on an ME composite [17], in which a permanent magnet and gear were used to generate an alternating magnetic field. This research demonstrated higher stability and sensitivity compared to coil sensors. In 2021, Lu et al. reported a gear speed sensor based on a FeCoSiB/PZT ME composite [18], which exhibited near-linear response characteristics in the speed range of 10 to 600 revolutions per minute (rpm). These studies focused on room temperature conditions but lacked tests on the response characteristics and reliability over a wider temperature range. In 2023, Zhao et al. reported a PZT/Metglas flexible ME sensor that works stably from 25 °C to 330 °C [19]. The ME sensor showed excellent and stable response capability at room temperature, but experienced a 61.8% decrease in ME coefficient at 330 °C. Additionally, this work did not explore the performance variation of the ME composite under subzero temperature conditions and lacked theoretical simulation support for environmental temperature changes.

In this work, we propose an ME tachometer based on a Metglas/PZT/Metglas composite, which operates stably over a wide temperature range from −70 °C to 160 °C. To comprehensively evaluate its performance, the response characteristics of the AC magnetic field under different frequencies and DC bias were measured and simulated. In the experiments, we used permanent magnets and a DC motor to simulate the rotational motion of gears, and positioned the tachometer parallel to the magnet’s rotation plane. By adjusting the distance and orientation of the ME tachometer precisely, we recorded the changes in voltage output. The results show thats when the tachometer is positioned close to the DC motor, the output voltage is significantly enhanced. In the speed range of 480 to 1260 rpm, the tachometer’s measured values closely match the preset speed values, demonstrating good measurement accuracy. Additionally, we tested the ME coefficients in the temperature range from −70 °C to 160 °C. Specific frequency points (30 Hz, 60 Hz, 90 Hz) and temperature points (−50 °C, 20 °C, 150 °C) were selected to analyze the variation trend of the ME tachometer’s response capability and the experimental waveforms. The results show that the proposed ME tachometer has reliable performance in extreme temperature environments, providing broad prospects for industrial speed detection and other application scenarios.

## 2. Design and Fabrication

**Figure 1:**
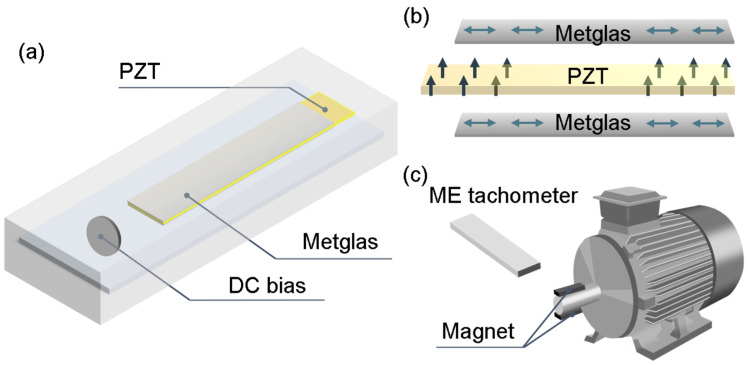
(**a**) Internal structure of the ME tachometer; (**b**) Working principle of the ME composite; (**c**) Schematic of the speed testing system.

We propose a sandwich-structured ME composite, which consists of three magnetostrictive Metglas amorphous ribbons (17 mm × 6 mm × 0.025 mm)/piezoelectric PZT (20 mm × 6 mm × 0.5 mm)/three magnetostrictive Metglas amorphous ribbons (17 mm × 6 mm × 0.025 mm). Interface effects play a critical role in the design of composite materials, especially in the contact and coupling methods between different materials [20,21,22]. In high-temperature environments, due to the differences in thermal expansion coefficients, the material interface may experience significant stress, which can become exacerbated during temperature variations, leading to interface failure such as micro-cracks or delamination, thus affecting the ME performance of the composite. By optimizing the thermal stability of the adhesive and improving the interface coupling between piezoelectric phase and magnetostrictive phase, the mechanical and electromagnetic performance of the ME composite under temperature variations can be effectively enhanced. High-temperature epoxy resin adhesive provided by 3M, Minnesota Mining and Manufacturing Company (Saint Paul, MN, USA) was used to couple the different phases. The ME composite was then set at 50 °C environment for 6 h to ensure the two phases were coupled tightly and expel air bubbles in the epoxy resin. As shown in Figure 1a, a permanent magnet providing the DC bias and the ME composite were enclosed in a 3D printed resin box. The ME effect refers to the phenomenon in which ME materials generate electrical signals under an external magnetic field, based on the “magneto-elastic-electric” coupling between the two phases [23] as illustrated in Figure 1b. When the ME composite is placed in an AC magnetic field, the magnetostrictive Metglas, which is magnetized along its length, generates strain. This strain is sensed by the piezoelectric PZT, which is polarized in the thickness direction, and it outputs induced charge, completing the conversion from a magnetic to electrical signal. For ME composites such as PZT/Metglas, the coupling effect between the magnetostrictive and the piezoelectric phases can be expressed by Equation (1):

(1) $$\frac{dE}{dH}=k_{1}k_{2}\cdot n\left(1-n\right)\cdot \frac{dS}{dH}\times \frac{dE}{dS}$$

dE/dH represents the ME coefficient of the composite; dS/dH and dE/dS represent the magnetostrictive effect and piezoelectric effect, respectively; n and 1−n represent the volume fraction of two phases; and $k_{1}$ and $k_{2}$ represent the proportional coefficients for the weakening caused by the interaction between the two phases. As shown in Figure 1c, two permanent magnets with opposite polarities are placed symmetrically on the motor’s rotation axis. When the motor rotates at the set frequency, the permanent magnets rotate accordingly, generating an oscillating magnetic field with the same rotation frequency. This alignment ensures that the magnetization direction of the Metglas is parallel to the plane where the rotation direction of the permanent magnet is located, optimizing deformation and maximizing the transfer of deformation to the piezoelectric phase, achieving the highest ME response and output voltage. The signal acquisition card provided by National Instruments was used to receive the output signal from the ME composite, which was then transmitted to the upper computer for Fast Fourier Transform (FFT), enabling the analysis of the time-domain signal regularity for verification.

## 3. Simulation of Extreme Temperature Environments

**Figure 2:**
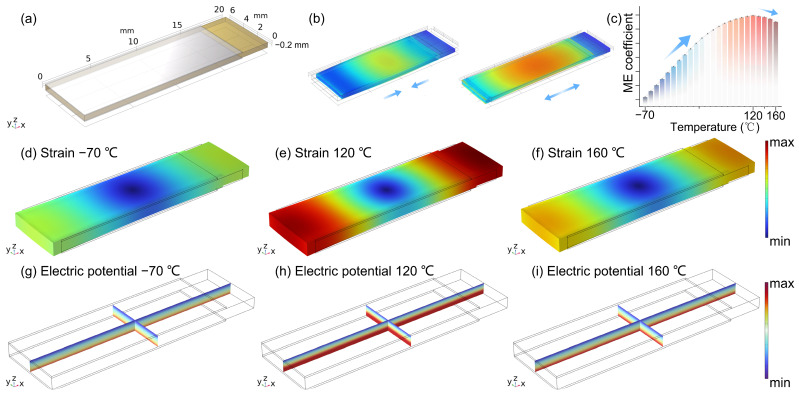
(**a**) Geometric model of the ME composite in COMSOL Multiphysics; (**b**) Schematic of the length resonance mode of the ME composite; (**c**) Simulation results of the variation of the ME coefficient with temperature; (**d**–**f**) Strain distribution of the ME composite at −70 °C, 120 °C, and 160 °C; (**g**–**i**) Electric potential distribution in the ME composite at −70 °C, 120 °C, and 160 °C.

To investigate the ME response of the composite in extreme temperature environments, we performed simulations using COMSOL Multiphysics 6.0. During the simulation, the electrostatic, magnetic field, and solid heat transfer modules were employed. In addition to coupling electric and magnetic fields, the thermoelectric module was used to couple the temperature field with the magnetic field, simulating environmental temperature variations. Based on the material properties, temperature-dependent coefficients such as thermal conductivity, the dielectric constant, and magnetostrictive coefficients were defined in the “Materials” section. As shown in Figure 2a, a geometric model was created according to the thickness and dimensions of the ME composite and assigned the corresponding material properties. For the sandwich-structured ME composite, its primary operating mode is the length-resonant mode [24], as shown in Figure 2b. The stress and deformation in the ME composite were mainly concentrated along the length during operation. The simulation results show that the resonance frequency closely matches the measured value (77 kHz), and the distribution of parameters such as electric potential and magnetic flux density at room temperature aligns with expectations, confirming the scientific validity of the constructed model.

Next, we coupled the temperature field and calculated the variation in the composite’s ME response as the temperature changed. The ME composite typically exhibits the maximum response at the resonance frequency [25], and when the environmental temperature or input intensity changes, the largest rate of change also occurs at the resonance frequency. To facilitate observation, we selected the resonance frequency for further calculations and analysis. The starting temperature was set to −70 °C, with increments of 10 °C, and the temperature gradually rose to 160 °C to observe the changes in the composite’s ME coefficient. The results, shown in Figure 2c, indicate that the ME coefficient increases with temperature up to 120 °C, then begins to decrease slowly. This is likely due to the mismatch in the coefficients of thermal expansion, which reduces the interface coupling efficiency and leads to material performance degradation [26]. Changes in stress can affect PZT’s strain and electric field response [27], impacting its coupling effect with Metglas. In extreme environments, long-term mechanical stress and thermal expansion cycles can also cause material fatigue, resulting in strain failure or poor electrode contact [22]; the stability and repeatability of the tachometer will be challenged. By further optimizing material selection, structure design, temperature control, stress management, and interface engineering, the negative impact of thermal expansion and mechanical stress on the ME composite can be minimized, thereby enhancing its reliability and precision in extreme environments. The strain in Figure 2d,e and the electric potential distributions in Figure 2g–i show a consistent trend. At 120 °C, the most intense colors in the distribution schematics represent the highest operating strength of the ME composite. For better visualization, the z-axis display ratio of the model was set to twice its original size. These simulation results provide theoretical foundation for subsequent experiments.

**Figure 3:**
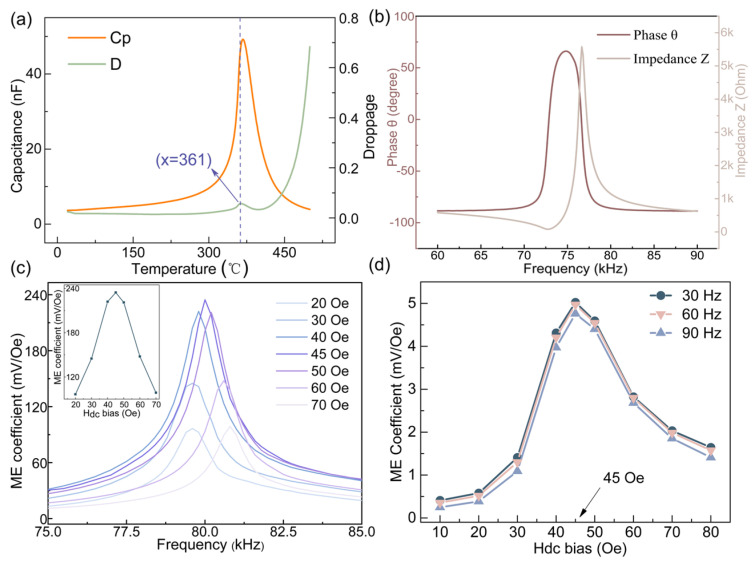
(**a**) Capacitance and dielectric loss of PZT as a function of temperature; (**b**) Impedance spectrum and phase angle of the ME composite; (**c**) Variation of the ME coefficient of the ME composite with DC bias magnetic field in the range of 20–70 Oe; (**d**) Variation of the ME coefficients of three characteristic frequencies with increasing temperature.

The capacitance and dielectric loss of PZT were tested with a temperature-dependent dielectric spectrometer to verify that the selected PZT can function properly in the high-temperature environment required for this work. The results are shown in Figure 3a. The properties of the selected PZT begin to change at 361 °C, meeting the experimental requirements. Based on the fabrication method of the ME composite mentioned above, the selected PZT coupled with the magnetostrictive phase, and the properties of the ME composite were tested. The ME coefficient is an important indicator for evaluating the ME conversion ability of the ME composite. To facilitate measurement and comparison, we selected the ME voltage coefficient ($a_{V}$, mV/Oe) to represent the strength of the ME effect. The magnitude of DC bias significantly affects the ME coefficient; we varied the DC bias by adjusting the DC current source applied to the Helmholtz coil. To determine the optimal DC bias value, the variations of the ME coefficient with DC bias at resonant frequency were observed. The reason for choosing this resonance frequency is that at this frequency, the variation amplitude of the ME coefficient is more pronounced, which facilitates accurate measurement and analysis.

## 4. Characterization of the ME Composite and ME Tachometer

The Agilent 4294A Impedance analyzer (Santa Clara, CA, USA) was used to test the phase angle and impedance spectrum of the ME composite, as shown in Figure 3b. In this work, the resonant frequency of the selected ME composite is 77 kHz. Since the ME coupling effect is strongest at the resonant frequency, it provides higher sensitivity, making it the operating frequency for many sensors. However, the responsiveness near the resonant frequency changes quickly, making it unsuitable for applications such as speed measurements in complex environments; under these conditions, the quasi-static responsiveness of the ME composite is more stable. Compared to traditional tachometers, ME tachometers have a broader frequency response range and can provide more reliable performance under extreme conditions. The ME composite in this work has a quasi-static frequency response exceeding 10 kHz, which can cover the frequency range of most motors while maintaining stable ME conversion coefficients. Next, the dynamic signal analyzer was employed to explore the variation of resonance frequency and ME coefficient with DC bias, as shown in Figure 3c. The results show that when the DC bias increases from 20 Oe to 70 Oe, the ME coefficients first increase and then decrease. Simultaneously, the resonant frequency shows a slight dispersion with changes in the DC bias. This phenomenon can be understood through the behavior of magnetostrictive material, which is closely related to the applied DC bias magnetic field. The magnetostrictive coefficient of Metglas exhibits nonlinear changes under an applied DC magnetic field [19]. As the value of DC bias increases, the magnetic domains in the material gradually reorient. Initially, this results in a gradual enhancement of the magnetostrictive effect because the alignment of magnetic domains increases the response to the external magnetic field. As the applied bias magnetic field strengthens, the magnetostrictive coefficient of the material increases until it reaches a critical point (the optimal DC bias) [28,29]. At this value, the magnetostrictive material reaches its maximum magnetostrictive coefficient, resulting in the strongest ME coupling effect and the highest ME coefficient.

In ME composites, the piezoelectric response of PZT and the magnetostrictive response of Metglas are coupled through their interface. As the DC bias magnetic field increases, the strain in the magnetostrictive material increases. More strain is transferred to the piezoelectric phase, which generates more induced charge. This enhances the ME effect and improves the ME coefficient. However, when the DC bias magnetic field exceeds a critical value, the coupling effect between the magnetostrictive and piezoelectric responses gradually weakens due to several reasons: a larger DC bias magnetic field may cause the magnetostrictive coefficient of Metglas to saturate, making it unable to further enhance the strain; when the applied DC bias is too large, the lattice structure of the Magnetostrictive material may distort or be damaged, which reduces its response ability in the magnetic field. As shown in Figure 3c, at the DC bias of 45 Oe, the composite’s ME response is maximized, and the ME coefficient reaches its highest value. Beyond 45 Oe, the magnetostrictive and piezoelectric responses begin to decrease, leading to a decline in the ME coefficient. In this work, the optimal DC bias was tested by gradually applying different strengths of the DC bias magnetic field using an electromagnet. The value of the optimal DC bias depends on factors such as the composite’s thickness, types, and polarization method. Different types and sizes of magnetostrictive materials have different optimal bias points [30].

Through comprehensive analysis, we determined that the optimal DC offset value for the ME composite is 45 Oe. Under this experimental condition, the ME coefficient of the proposed ME composite reaches as high as 237 mV/Oe. To further verify the accuracy of the optimal DC bias, we selected three characteristic frequencies (30 Hz, 60 Hz, 90 Hz) to observe the variation of the ME coefficient with DC bias. As shown in Figure 3d, the results show that at these three characteristic frequencies, the ME coefficients first increase and then decrease with increasing DC bias, reaching the maximum value when the DC bias is 45 Oe. This trend is consistent with that observed at the resonant frequency, confirming that 45 Oe is the optimal DC bias value for the proposed ME composite. Finally, a KANETEC TM-901EXP Gauss meter (Tokyo, Japan) was used to accurately measure the strength of the magnetic field produced by the permanent magnet and fixed it at a position corresponding to 45 Oe. The factors influencing the stability and repeatability of the ME coefficient are multifaceted, including the material’s temperature dependence, interface effects, fatigue effects, control of experimental condition, and external environmental influences. By optimizing material selection and interface design, controlling experimental conditions, and enhancing equipment precision, the stability and repeatability of the ME coefficient can be improved, ensuring the reliable performance of the ME tachometer across different temperatures.

**Figure 4:**
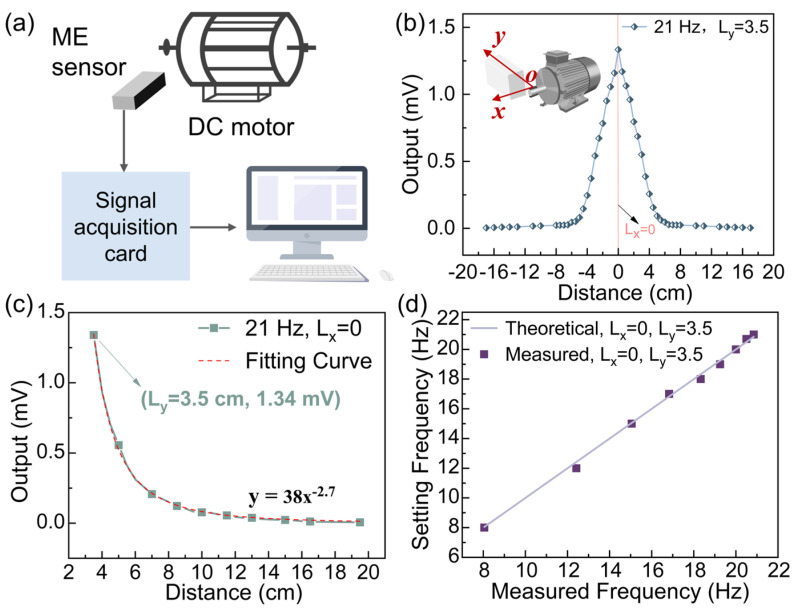
(**a**) Schematic of gear speed test system; (**b**) Variation of output voltage with distance along the x-axis; (**c**) Variation of output voltage with distance along the y-axis; (**d**) Comparison of measured and preset rotational speeds.

The speed measurement system was set up as shown in Figure 4a. The DC motor has significant advantages over other types of motors in terms of stability. Its speed and output characteristics can be precisely adjusted by controlling the voltage or current, ensuring high stability during long-term operation. Due to its relatively simple structure and low noise level, the DC motor helps minimize interference with the test signals, further improving the accuracy and stability of the measurement results. Therefore, we chose the DC motor for our experiments to ensure the reliability and precision of the test results. The ME tachometer was placed next to the DC motor in the correct orientation. When the motor ran, it drove a pair of permanent magnets. The ME tachometer sensed the AC magnetic field signal and generated an electrical signal. The signal acquisition card received this electrical signal and transmitted it to the upper computer software for subsequent processing. FFT was applied to the upper computer to analyze the collected data. To define the spatial coordinate system, the following reference was established: the center of the permanent magnet was defined as the origin (0, 0); the direction along the shaft of DC motor was taken as the *x*-axis, and the direction perpendicular to the shaft was defined as the *y*-axis. In the experiment, a pair of 200 mT magnets was used, with the motor’s rotational frequency set to 21 Hz.

The rotational speed was calculated to be 1260 rpm using Equation (2), where f is the frequency and N is the rotational speed, demonstrating the relationship between the rotational speed and the signal frequency.

(2) N rpm=f·60

First, the ME tachometer was translated along a straight line at y=2, within the range of −17 cm to 17 cm on the x-axis, while its output voltage was recorded. As shown in Figure 4b, when the ME tachometer was positioned at (0, 2), the output voltage reached the maximum value of 1.34 mV. As the distance between the tachometer and the permanent magnet increased, the output voltage gradually decreased, showing good symmetry and regularity, indicating the system’s stable and predictable response. Subsequently, the ME tachometer was moved from 2 cm to 18 cm along the y-axis. It is noteworthy that this direction of movement is consistent with the propagation direction of the electromagnetic wave. According to the theory of electromagnetic wave propagation, the magnetic field strength in the near-field region decreases with distance following a cubic decay law (${1/r}^{3}$) [31,32]. As shown in Figure 4c, the output voltage decays proportionately with ${1/r}^{2.7}$, fitting well ($R^{2}=0.99$), which aligns with the electromagnetic wave attenuation characteristics. The slight deviation is attributed to the volume effect [33]. Finally, to evaluate the accuracy of the tachometer, we fixed the tachometer at (0, 3) and set the motor speed to vary from 8 to 21 Hz (480 to 1260 revolutions per minute). The results show that the measured speed of the tachometer is in good agreement with the preset value, confirming its reliability and high-precision performance across a wide speed range.

## 5. Performance Testing in Extreme Temperature Environments

**Figure 5:**
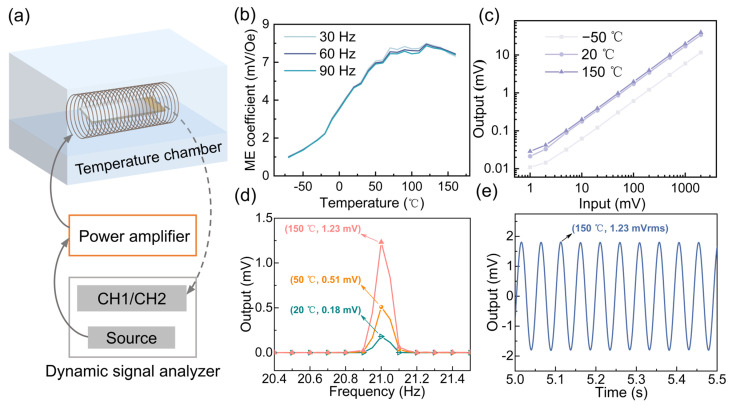
(**a**) Schematic of the extreme temperature measurement system; (**b**) Variation of the ME coefficient with characteristic frequencies over the temperature range of −70 °C to 160 °C; (**c**) Linearity of the ME composite at characteristic frequencies for the selected temperatures; (**d**) Voltage output of the ME tachometer at characteristic temperatures; (**e**) Output waveform of the ME tachometer during operation.

Based on the validation of the ME tachometer’s ability to accurately measure gear speed, a professional temperature chamber was used to create the required temperature conditions, as shown in Figure 5a. The ME composite was placed inside a coil with an inner diameter of 5 cm and a length of 10 cm. An alternating signal was generated by a dynamic signal analyzer, amplified by a power amplifier, and applied to the coil. The ME composite sensed the alternating magnetic field, converted it into charge signals, and transmitted these signals to the analyzer.

The performance of the ME composite was evaluated over a temperature range of −70 °C to 160 °C. As shown in Figure 5b, the ME coefficient increased with temperature up to 120 °C, after which it began to slowly decrease. These trends are consistent with the simulation results shown in Figure 2c. At subzero temperatures, the magnetic domain structure is less stable than at room temperature, weakening the magnetic field’s effect on the material, thus affecting the ME effect [34]. As temperature increases, the ME coefficient of the composite steadily increases due to the gradual enhancement of the magnetization in the magnetostrictive phase and the improvement of the polarization effect in the piezoelectric phase. However, after 120 °C, the ME coefficient starts to decrease as the temperature increases. This may be due to several factors: first, the magnetization of the magnetostrictive phase gradually weakens, reducing its response to external magnetic fields. Additionally, at high temperatures, the polarization effect of the piezoelectric phase diminishes because increased lattice rigidity limits the movement of dipoles [34]. Additionally, temperature rise may cause changes in the interface stress between the magnetic and piezoelectric phases, and the weakening of the coupling effect at the interface could also contribute to the decrease in the ME coefficient. Thermal expansion effects may cause interface stress changes between the two phases, affecting overall performance. The variations in the material’s dielectric constant, conductivity, and crystal structure further contribute to fluctuations in the ME coefficient [35]. Furthermore, the significant impact of temperature on the response of ME composites may be closely related to the spring-hardening effect in piezomagnetic material and the spring-softening effect in piezoelectric material [36], with the effect of piezomagnetic materials being more significant. The spring-hardening effect refers to the phenomenon where the Young’s modulus (stiffness) of magnetostrictive materials changes during the magnetization process. This hardening effect can influence the strain of the magnetostrictive material, thereby altering the overall response characteristics of the ME composite.

Another possible reason is the effects of epoxy resin adhesive performance in high-temperatures: Although the rated maximum operating temperature of the adhesive we used is higher than the highest in our experiments, the adhesive may have slight softening, which could lead to a reduction in its bonding strength, weakening the interface coupling between PZT and Metglas. When the coupling efficiency weakens, the deformation caused by the magnetic field will be transmitted less to PZT, affecting the overall ME coupling. ME coefficient α of the composite can be expressed as Equation (3) [28]:

(3) $$a=\frac{\partial P}{\partial H}=k_{c}\frac{\partial P}{\partial s}\frac{\partial s}{\partial H}$$

where H is the applied magnetic field, S is the mechanical strain at the phase interface, and P is the polarization generated within the piezoelectric phase. The temperature-induced change in interface coupling primarily impacts $k_{c}$ (the coupling coefficient that describes the elastic coupling between the two phases). The change in $k_{c}$ is related to factors such as the adhesive’s properties, ratio, and thickness. When the temperature exceeds 120 °C, the softening of the adhesive will reduce $k_{c}$. This change is ultimately reflected in the variation of the ME coefficient. Although this change does not represent a drastic deterioration in the composite’s performance, it does have some impact on the stability of the ME composite.

Next, the linearity of the ME composite’s output voltage at a specific frequency (30 Hz) was assessed. Representative temperatures of −50 °C, 20 °C, and 150 °C were selected, and at each temperature point, the applied voltage to the coil was gradually increased from 1 mV to 2000 mV. The output voltages of the ME composite were measured as the input voltage changed. As shown in Figure 5c, the output voltages of the ME composite increased steadily as the input voltage increased, demonstrating excellent linearity. Furthermore, at −50 °C, 20 °C, and 150 °C, the output voltage of the ME composite increased successively. Next, the performance of the ME tachometer in measuring rotational speed from 70 °C to 160 °C was tested. The DC motor was placed outside the variable temperature chamber, while the ME tachometer was placed inside the chamber to directly assess its performance under different temperature conditions. The motor speed was set to 1260 rpm (21 Hz), simulating typical operating conditions. As shown in Figure 5d, at 20 °C, 50 °C, and 150 °C, the voltage outputs of the ME tachometer were 0.18 mV, 0.51 mV, and 1.23 mV, respectively. To quantify the performance of the ME tachometer more scientifically, the signal-to-noise ratio (SNR) was used as the evaluation metric. Based on Equation (4), the SNR values at 20 °C, 50 °C, and 150 °C are 49.77 dB, 49.54 dB, and 51.7 dB, respectively.

(4) $$SNR(dB)=20\times {log}_{10}(\frac{V_{signal}}{V_{noise}})\;$$

The main sources of noise affecting the ME tachometer include electromagnetic interference (EMI), mechanical vibration noise, and signal processing noise. EMI mainly arises from surrounding electrical equipment and communication systems, which is particularly pronounced in high-frequency environments. Mechanical vibration noise is inevitable in dynamic measurement applications, especially in environments involving gears or other moving parts. This can cause displacement or deformation of the sensor or mounting, affecting the stability of the measurement signal. Signal processing noise can be introduced during amplification, filtering, and analog-to-digital conversion (ADC). Although thermal noise theoretically increases with temperature, our experimental results show that the SNR remains around 50 dB at 20 °C, 50 °C, and 150 °C. This is likely due to the stable electrical and magnetic properties of PZT and Metglas in this temperature range, and the signal processing system effectively mitigates the impact of thermal noise. In contrast, EMI and mechanical vibration noise may be more dominant sources of noise [37], with their impact closely related to the industrial environment and operational frequency. The observed stability of the SNR suggests that the ME tachometer maintains reliable performance across different temperature and frequency conditions.

The results show that the ME tachometer is capable of maintaining good measurement performance in extreme temperature environments, accurately capturing its speed signal, and its high SNR values ensure stability in complex temperature and electrical environments. Furthermore, the output voltage waveform of the ME tachometer was analyzed in detail, and the measured output waveform when the motor was operating at 1260 rpm (21 Hz) is shown in Figure 5e. The waveform presents a regular sinusoidal signal, indicating that the tachometer can measure the motor speed stably. During the experiment, the output waveform maintained good stationarity, with no obvious noise or waveform distortion, indicating that the ME tachometer has good measuring accuracy and anti-interference capability. In addition, although high temperatures may result in some electrical noise or temperature drift, the experimental waveform showed no significant interference nor nonlinear errors, proving the stability and reliability of the ME tachometer in complex environments.

## 6. Conclusions

The proposed tachometer based on the Metglas/PZT/Metglas ME composite successfully achieves high-precision measurements in a wide temperature range (−70 °C to 160 °C). Through detailed experimental tests, we systematically evaluated the response characteristics of the ME tachometer under different frequencies, DC bias, and temperature conditions. The results show that the ME tachometer has good measurement consistency and high precision in the speed range of 480 to 1260 rpm, especially in extreme temperature environments, and can maintain stable performance, proving its excellent environmental adaptability and reliability. Through the analysis of the performance of the ME composite, this work explores the ME coefficient under different temperature conditions. The tachometer’s high SNR and good anti-interference capability enable it to meet the harsh environmental requirements of industrial applications, especially in high-temperature testing. In addition, the non-contact measurement method allows the ME tachometer to have a longer service life and lower maintenance costs under complex conditions such as high temperatures.

Although this work provides an initial design for ME tachometers applied in wide temperature ranges, several aspects still need further exploration. First, interface engineering and material optimization are key directions for future research. Especially under extreme temperature conditions, the impact of interface effects on the composite’s performance cannot be overlooked. Future works could focus on introducing buffer layers or other optimization layers to reduce thermal expansion mismatch, ensuring stable coupling between the two phases of the ME composite, and improving the long-term stability of the ME composite [20]. Additionally, regarding the impact of temperature changes on the performance of the ME tachometer, temperature compensation algorithms could be designed in future work to further enhance stability and precision under varying temperature conditions. The implementation of such compensation mechanisms would significantly improve the reliability of ME tachometers, particularly in extreme temperature environments [38,39]. The precision of the temperature control system is also important for future improvement. To minimize the impact of temperature gradients on the ME composite’s performance, future research could adopt more precise temperature control equipment to ensure uniform distribution, further guaranteeing the accuracy and reliability of measurement results [40]. Lastly, as we validated the performance at low temperatures, future work will explore how to maintain the ME tachometer’s stability and performance across an even broader temperature range. With the optimization of materials and manufacturing processes, the tachometers based on ME composites are expected to expand into more industrial fields, including aerospace, automotive, robotics, and other high-demand environments.

## Data Availability

Data are contained within the article.

## Abbreviations

The following abbreviations are used in this manuscript:

ME	Magnetoelectric
rpm	Revolutions per minute
SNR	Signal-to-noise ratio
FFT	Fast Fourier transform
